# Residual risk of HIV, HBV, and HCV transmission through blood transfusion in Borgou, Benin, 2023–2025

**DOI:** 10.3389/frph.2026.1861977

**Published:** 2026-06-04

**Authors:** Kamel-Dine Djaliri, Adolphe Adjanohoun, Pamphile Aguessy, Victorien Dougnon, Haziz Sina, Lamine Baba-Moussa

**Affiliations:** 1 Laboratory of Biology and Molecular Typing in Microbiology, Faculty of Sciences and Technology, University of Abomey-Calavi, Cotonou, Benin; 2 Research Unit in Applied Microbiology and Pharmacology of Natural Substances, Laboratory of Applied Microbiology, Polytechnic School of Abomey-Calavi, Cotonou, Benin; 3 National Blood Transfusion Agency (ANTS), Cotonou, Benin

**Keywords:** Benin, blood donors, blood safety, hepatitis B virus, hepatitis C virus, HIV, incidence, residual risk

## Abstract

**Background:**

Blood transfusion is a cornerstone of modern healthcare, particularly in maternal and pediatric care. However, it continues to carry a residual risk of transmitting transfusion-transmissible infections (TTIs), especially human immunodeficiency virus (HIV), hepatitis B virus (HBV), and hepatitis C virus (HCV). Quantifying this residual risk is critical for optimizing transfusion safety, particularly in resource-limited settings. This study aimed to estimate the residual risk of HIV, HBV, and HCV transmission among blood donors in the Borgou department of Benin.

**Methods:**

A retrospective cohort study of repeat donors was conducted from January 2023 to December 2025 among repeat blood donors in Borgou. Serological screening was performed using enzyme-linked immunosorbent assay (ELISA) with Bio-Rad® kits. Residual risk was estimated using the Schreiber model based on incidence/window period methodology. Data were analyzed using SPSS software, and associations were assessed using the Chi-square test, with statistical significance set at *p* < 0.05.

**Results:**

A total of 11,113 repeat donors were included, with a marked male predominance (male-to-female ratio: 14.14). The majority were aged 18–30 years (72%) and were students (54%). Overall, 145 incident infections were identified. Incidence rates per 100,000 donor-years were 5.98 for HIV, 350.28 for HBV, and 77.84 for HCV. No significant association was observed between HIV incidence and sociodemographic characteristics. In contrast, HBV and HCV incidences were significantly associated with age (*p* < 0.001 and *p* = 0.03, respectively), with higher rates observed among donors aged 18–24 years. Estimated residual risks were 1 in 277,000 donations for HIV, 1 in 1,862 for HBV, and 1 in 7,070 for HCV.

**Conclusion:**

Although the residual risk of TTIs in Borgou is lower than that reported in several sub-Saharan African settings, it remains substantially higher than in high-income countries. These findings highlight the urgent need to strengthen donor selection strategies, enhance targeted health education, and implement more sensitive screening technologies, such as nucleic acid testing, to further improve blood safety.

## 1 Introduction

Blood transfusion is a critical component of modern healthcare, playing a pivotal role in the management of life-threatening conditions such as severe anemia, acute hemorrhage - particularly in obstetric emergencies - and hematological disorders, including sickle cell disease. Globally, an estimated 118.5 million blood donations are collected each year, with nearly 40% originating from high-income countries, which represent only 16% of the world's population (1). This disparity highlights major inequities in blood availability. In low-resource settings, especially in sub-Saharan Africa, obstetric complications account for up to 30%–50% of transfusion indications, and inadequate access to safe blood significantly contributes to maternal mortality, which remains as high as 545 deaths per 100,000 live births in some countries of the region (1, 2).

According to the World Health Organization, blood transfusion is a life-saving therapeutic intervention aimed at restoring circulating blood volume and correcting deficiencies in specific blood components (1). Despite its undeniable clinical benefits, transfusion therapy carries inherent risks, notably the transmission of infectious agents. Over the past decades, substantial progress has been achieved in enhancing transfusion safety. Globally, 79 countries now collect over 90% of their blood supply from voluntary non-remunerated donors, a strategy associated with lower infection risk (1). In addition, systematic screening of major transfusion-transmissible infections (TTIs)- including human immunodeficiency virus (HIV), hepatitis B virus (HBV), hepatitis C virus (HCV), and syphilis-has significantly reduced transmission risks (3–4). However, in sub-Saharan Africa, the prevalence of these infections among blood donors remains comparatively high, with reported ranges of 1%–3% for HIV, 5%–15% for HBV, and 1%–7% for HCV, depending on the setting (5, 4).

Despite these advances, transfusion safety is not absolute. A residual risk persists, primarily due to the serological window period during which infections remain undetectable and to the intrinsic limitations of screening techniques. In high-income countries, this residual risk has been reduced to extremely low levels-estimated at approximately 1 in 1–2 million donations for HIV, 1 in 300,000–1,000,000 for HCV, and 1 in 200,000–500,000 for HBV-largely due to the implementation of nucleic acid testing (NAT) (6). In contrast, in many African settings, the residual risk remains substantially higher, particularly for HBV, reflecting both higher incidence rates and limited access to advanced screening technologies.

In sub-Saharan Africa, the combination of high demand for blood products and limited healthcare infrastructure further complicates efforts to mitigate this risk. In Benin, national transfusion safety strategies aligned with World Health Organisation recommendations have been implemented, including promoting voluntary blood donation and adopting fourth-generation serological assays (1, 2). However, national data indicate that transfusion-transmissible infections remain a concern, with HBV prevalence among donors often exceeding 8%–10% in some West African contexts (5).

However, the lack of robust epidemiological surveillance systems for blood donors, coupled with limited post-transfusion recipient follow-up, hampers accurate estimation of residual risk. To date, no study has specifically quantified the residual risk of HIV, HBV, and HCV transmission among blood donors in the Borgou department. This data gap represents a major limitation for evidence-based evaluation of transfusion safety and the design of targeted preventive strategies. What is the level of residual risk of HIV, HBV, and HCV transmission associated with blood transfusion in the Borgou department of Benin? The objective of this study is to estimate the residual risk of HIV, HBV, and HCV transmission among blood donors in the Borgou region, to generate evidence to inform and strengthen transfusion safety strategies.

## 2 Material and methods

### 2.1 Study design and setting

A retrospective cohort study of repeat donors with an analytical component was conducted over three years from January 2023 to December 2025 in the Borgou department of Benin. This design was selected to enable both the estimation of incidence rates and the identification of factors associated with seroconversion among blood donors, in line with established approaches in observational epidemiology.

### 2.2 Study population and sampling

The study population consisted exclusively of repeat blood donors, defined as individuals who had donated blood at least twice during the study period. This definition is recommended in transfusion epidemiology because it allows the identification of incident infections through the detection of seroconversion between successive donations (7). Donor frequency was categorized into three groups: first-time donors (first-ever donation), regular donors (≥2 donations), and loyal donors (≥4 donations per year).

- Inclusion criteria:

Included in the study were repeat donors who had made at least two donations during the study period and were declared eligible for blood donation following the pre-donation medical interview. Participants were aged between 18 and 65 years, had a minimum body weight of 50 kilograms, and were apparently in good health, in accordance with standard blood donor selection criteria (8).

- Non-inclusion criteria:

Not included in the study were donors presenting a contraindication to blood donation and declared ineligible following the pre-donation medical interview, as well as first-time donors making their initial donation, since incidence estimation requires longitudinal follow-up.

### 2.3 Data sources and variables

Data were retrospectively collected from standardized transfusion service records, including pre-donation medical questionnaires, donor registry databases, and laboratory screening registers. Each donor was assigned a unique anonymized identification code, allowing longitudinal tracking of donations while ensuring confidentiality. The collected variables included sociodemographic characteristics such as age, sex, occupation, and donation site, as well as donation-related variables including the number and frequency of donations. In addition, biological data corresponding to serological results for human immunodeficiency virus (HIV), hepatitis B virus (HBV), and hepatitis C virus (HCV) were systematically recorded.

**Blood Sample Collection and Laboratory Procedures.** Screening for the main transfusion-transmissible infections, namely HIV, HBV, and HCV, was performed using fourth-generation ELISA techniques, in accordance with the international recommendations of the World Health Organization (9). The assays used were validated commercial kits (Bio-Rad), enabling the simultaneous detection of viral antigens and antibodies, thereby significantly reducing the serological window period and improving diagnostic sensitivity and specificity (10). However, in the absence of molecular testing (PCR/NAT), a residual window period persists, corresponding to the very early phase of infection during which both serological and antigenic markers remain undetectable. This limitation represents a potential source of underestimation of the residual transfusion risk.

- The GENSCREEN™ ULTRA HIV Ag-Ab kit, used for HIV screening, is a qualitative enzyme immunoassay designed for the simultaneous detection of HIV-1 p24 antigen and anti-HIV-1 antibodies (groups M and O), as well as anti-HIV-2 antibodies in human serum or plasma (11).
- The MONOLISA™ HBsAg ULTRA kit, used for HBV screening, is a one-step qualitative sandwich enzyme immunoassay intended for the detection of hepatitis B surface antigen (HBsAg) in human serum or plasma (12)
- The MONOLISA™ HCV Ag-Ab ULTRA V2 kit, used for HCV screening, allows the simultaneous detection of anti-HCV antibodies and core antigen in human serum or plasma (13).

The analytical performance of these assays, particularly in terms of sensitivity and specificity, is consistent with manufacturer specifications and supported by scientific literature on HIV, HBV, and HCV screening in blood transfusion settings (Bio-Rad Laboratories (9, 11, 12, 13).

For each series of assays, the controls provided in the reagent kits, along with internal controls prepared from samples with known results, are used to validate the results obtained.

All initially reactive samples were managed in accordance with national transfusion guidelines, including confirmatory procedures consisting of repeat testing on a new sample using Bio-Rad ELISA assays considered as reference methods. Donors were also informed, counselled, and referred when necessary for appropriate medical care.

### 2.4 Estimation of incidence and residual risk

The incidence rate of transfusion-transmissible infections was calculated among repeat donors as the number of newly identified seroconversion cases divided by the total person-time at risk, expressed in person-years. Seroconversion is defined as the transition from a seronegative status to a seropositive status between two consecutive donations. Person-time was estimated by calculating, for each donor, the interval in days between the first and last recorded donations during the study period and converting this duration into years by dividing by 365 (14). The residual risk of viral transmission through blood transfusion was then estimated using the incidence/window period model described by Schreiber et al. (6), which combines the observed incidence rate with the duration of the serological window period.

RR = Incidence   ×   Window period/365

**RR:** Residual risk.

**Incidence:** Ratio of the number of seroconversions among repeat donors during the study period to the total number of person-years (PY).

**PY:** Person-years, calculated by summing, for each donor, the intervals (in days) between the first and the last donation, then dividing the total by 365.

In the absence of nucleic acid testing, standard serological window periods were applied based on published data, with estimated durations of 22 days for HIV, 56 days for HBV, and 66 days for HCV (15).

However, this methodology, although widely used in resource-limited contexts, has a limitation. The use of the incidence/window period model is an indirect approach that is highly dependent on window period estimates derived from the literature. The absence of molecular screening (TAAN/NAT) is likely to result in an underestimation of the residual risk, particularly for infections in the very early phase.

### 2.5 Data processing and statistical analysis

Data were entered into Microsoft Excel following prior coding and subsequently subjected to rigorous cleaning procedures, including consistency checks, detection of outliers, and elimination of duplicate entries. Statistical analyses were performed using IBM SPSS Statistics version 20.0. Descriptive statistics were used to summarize the characteristics of the study population, with categorical variables expressed as frequencies and proportions, and continuous variables presented as means with standard deviations or medians with interquartile ranges, depending on the distribution of the data. Incidence rates were calculated per 100,000 person-years to ensure comparability with existing studies. Associations between categorical variables were assessed using the Chi-square test or Fisher's exact test when appropriate. In addition, multivariate logistic regression analysis is performed to identify independent factors associated with seroconversion, with results expressed as odds ratios and their 95% confidence intervals. A *p*-value of less than 0.05 was considered statistically significant.

### 2.6 Ethical considerations

The study protocol was approved by the Ethics and Research Committee of the Institute of Applied Biomedical Sciences (CER-ISBA, approval No. 154 of December 22, 2022) and authorized by the National Blood Transfusion Agency, the regulatory authority responsible for transfusion services in Benin. Written informed consent was obtained from all participants before inclusion. Throughout the study, strict measures were implemented to ensure the confidentiality, anonymity, and secure handling of donor data, in accordance with internationally accepted ethical standards for biomedical research.

## 3 Results

### 3.1 Sociodemographic characteristics of repeat blood donors

During the study period (2023–2025), a total of 29,609 individuals donated blood, among whom 11,134 were identified as repeat donors. The donor population was predominantly male (93.4%, *n* = 10,399/11,134), corresponding to a male-to-female ratio of 14.14. The majority of donors were young adults, with 49.6% aged 18–24 years and 71.1% aged between 18 and 30 years, indicating a strongly youth-dominated donor base. Students represented the largest socio-professional group (54.2%, *n* = 6,034/11,134), followed by employees (18.2%, *n* = 2.025/11,134) and artisans (17.8%, 1,981/11,134). More than half of donations were collected through mobile blood collection teams (54.3%, *n* = 6,047/11,134), while 45.7% (*n* = 5,087/11,134) were performed at fixed sites. These findings are detailed in Table 1.

**Table 1:** Sociodemographic characteristics of repeat blood donors in Borgou (2023–2025).

Characteristic	N	%	95% CI
Sex (*n* = 11,134)			
Male	10,399	93.4	92.6–94.1
Female	735	6.6	5.9–7.4
Age group (years)			
18–24	5,528	49.6	48.9–50.3
25–30	2,396	21.5	20.7–22.3
31–44	2,422	21.7	20.9–22.5
45–64	788	7.1	6.6–7.6
Occupation			
Students	6,034	54.2	53.3–55.1
Employees	2,025	18.2	17.4–19.0
Artisans	1,981	17.8	16.7–19.1
Others	1,094	9.8	9.1–10.5
Donation site			
Mobile teams	6,047	54.3	53.2–55.4
Fixed sites	5,087	45.7	44.6–46.8

### 3.2 Annual donation frequency

**Figure 1:**
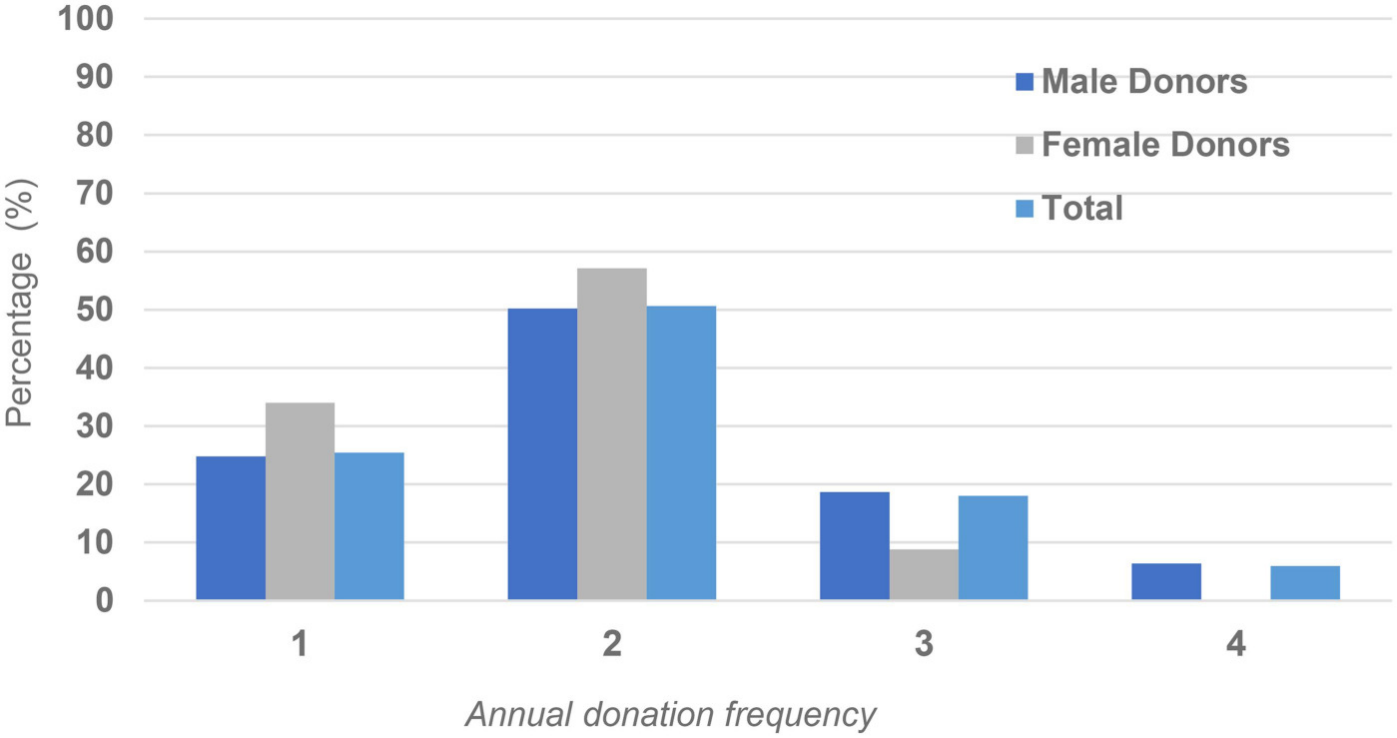
Annual frequency of blood donations among repeat donors in Borgou (2023–2025).

As illustrated in Figure 1, most repeat donors contributed two donations per year (50.63%, *n* = 5,637/11,134), followed by those donating once annually (25.42%, *n* = 2,830/11,134). Higher donation frequencies were progressively less common, with 18.00% (*n* = 2,005/11,134 donating three times per year and only 5.95% (*n* = 662/11,134) reaching four donations annually.

Male donors exhibited a higher proportion of individuals making three donations per year (18.66%, *n* = 1,940/10,399) compared with female donors (8.84%, *n* = 65/735). Conversely, for a frequency of two annual donations, female donors were more represented (57.15%, *n* = 420/735) than their male counterparts (50.17%, *n* = 5,217/10,399).

### 3.3 Incidence of HIV, HBV, and HCV among blood donors

A total of 145 incident infections were identified during the study period. Hepatitis B virus (HBV) accounted for the majority of cases (80.69%, *n* = 117/145), followed by hepatitis C virus (HCV) (17.93%, *n* = 26/145) and human immunodeficiency virus (HIV) (1.38%, *n* = 2/145). The overall incidence rates per 100,000 donor-years were 5.98 for HIV, 350.27 for HBV, and 77.84 for HCV, highlighting a substantially higher burden of HBV among repeat donors compared to the other viral markers. These data are summarized in Table 2.

**Table 2:** Incidence rates of HIV, HBV, and HCV among blood donors.

Virus	Incident cases (n)	%	Person-years (PY)	Incidence rate (per 100,000 PY)
HIV	2	1.38	33,402	5.98
HBV	117	80.69	33,402	350.27
HCV	26	17.93	33,402	77.84
Total	145	100	33,402	434.10

### 3.4 Incidence by sex and age group

As shown in Table 3, males accounted for 96.55% (*n* = 140/145) of all incident infections. All HIV cases occurred in male donors; however, no statistically significant association was observed between sex and HIV incidence. This Interpretation of HIV-specific associations is limited due to the very small number of cases. In contrast, age distribution showed a clear pattern, with the highest proportion of incident infections occurring in donors aged 18–24 years (63.45%, *n* = 92/145). This trend was consistent across HBV and HCV infections, where this age group accounted for 62.39% (*n* = 73/117) and 69.23% (*n* = 18/26) of cases, respectively. The association between age and incidence was statistically significant for HBV and HCV, indicating a higher risk of infection among younger donors.

**Table 3:** Distribution of HIV, HBV, and HCV incident cases by sex and age group among blood donors.

Variables	Total incident cases (*N* = 145) *n* (%)	HIV (*n* = 2) *n* (%)	*p*-value	HBV (*n* = 117) *n* (%)	*p*-value	HCV (*n* = 26) *n* (%)	*p*-value
Sex			**1**.**00**		**0**.**70**		**1**.**00**
Male	140 (96.55)	2 (100.0)		114 (97.44)		24 (92.31)	
Female	5 (3.45)	0 (0.0)		3 (2.56)		2 (7.69)	
Age group (years)			**0**.**65**		**0**.**001**		**0**.**03**
18–24	92 (63.45)	1 (50.0)		73 (62.39)		18 (69.23)	
25–30	29 (20.00)	0 (0.0)		24 (20.51)		5 (19.23)	
31–44	17 (11.72)	0 (0.0)		15 (12.82)		2 (7.69)	
45–64	7 (4.83)	1 (50.0)		5 (4.27)		1 (3.85)	

Bold values indicates any *p*-value less than 0.05 was considered statistically significant.

### 3.5 Incidence by socio-professional category and donation site

As illustrated in Table 4, students constituted the most affected socio-professional group, representing 66.21% (*n* = 96/145) of all incident cases, followed by artisans and other employees. Although students showed the highest absolute number of infections for HBV and HCV, no statistically significant association was observed between the socio-professional category and infection incidence. This situation may be explained by the non-negligible proportions of incident cases observed among artisans (13.78%; *n* = 20/145) and other employees (13.78%; *n* = 20/145).

**Table 4:** Distribution of HIV, HBV, and HCV incident cases according to socio-professional category and donation site.

Variables	Total incident cases (*N* = 145) *n* (%)	HIV (*n* = 2) *n* (%)	*p*-value	HBV (*n* = 117) *n* (%)	*p*-value	HCV (*n* = 26) *n* (%)	*p*-value
Socio-professional category			**1**.**00**		**0**.**66**		**1**.**00**
Students (pupils/university)	96 (66.21)	1 (50.0)		75 (64.10)		20 (76.92)	
Artisans	20 (13.79)	0 (0.0)		19 (16.24)		1 (3.85)	
Other employees	20 (13.79)	1 (50.0)		17 (14.53)		2 (7.69)	
Military/paramilitary	2 (1.38)	0 (0.0)		1 (0.85)		1 (3.85)	
Medical/paramedical staff	3 (2.07)	0 (0.0)		2 (1.71)		1 (3.85)	
Other occupations	4 (2.76)	0 (0.0)		3 (2.56)		1 (3.85)	
Donation site			**0**.**89**		**<0**.**001**		**0**.**48**
Mobile collection teams	**124** (**85.52)**	**1** (**50.0)**		**103** (**88.03)**		**20** (**76.92)**	
Fixed collection sites	**21** (**14.48)**	**1** (**50.0)**		**14** (**11.97)**		**6** (**23.07)**	

Bold values indicates any *p*-value less than 0.05 was considered statistically significant.

In contrast, the donation site showed a more marked effect. The majority of incident cases (85.52%, *n* = 124/145) were observed among donors recruited through mobile collection teams, compared with fixed donation centers. This difference reached statistical significance for HBV infections, suggesting a higher exposure risk among donors recruited via mobile strategies.

### 3.6 Residual transfusion risk

The estimated residual risk of transfusion-transmitted infections showed marked variation across viral markers. HIV presented the lowest residual risk, estimated at 3.61 × 10⁻⁶ (approximately 1 in 277,000 donations). HBV showed the highest residual risk at 5.37 × 10⁻⁴ (approximately 1 in 1,862 donations), while HCV presented an intermediate risk of 1.41 × 10⁻⁴ (approximately 1 in 7,100 donations). These results indicate that HBV remains the predominant residual transfusion risk in the study setting (Table 5).

**Table 5:** Residual risk of HIV, HBV, and HCV transmission.

Virus	Incidence (n)	Person-years (PY)	Window (days)	RR	Risk (1 in *n* donations)
HIV	2	33,402	22	3.61 × 10⁻⁶	1/277,000
HBV	117	33,402	56	5.37 × 10⁻⁴	1/1,862
HCV	26	33,402	66	1.41 × 10⁻⁴	1/7,092

Window periods used for calculations are based on standard durations for 4th generation ELISA assays (HIV: 22 days; HBV: 56 days; HCV: 66 days).

## 4 Discussion

Blood safety remains a central and continuous concern for transfusion services worldwide, relying on the implementation of effective hemovigilance systems that ensure the traceability, monitoring, and prevention of transfusion-transmissible infections. In the Borgou Blood Transfusion Service (STS-B) in Benin, several strategies are implemented to strengthen transfusion safety, including the recruitment of voluntary non-remunerated donors, systematic pre-donation medical screening, and the biological qualification of all blood units using high-performance serological assays. These measures are consistent with international recommendations aimed at minimizing transfusion risks (1, 2). Despite these interventions, residual risk persists, making its quantification essential for evaluating the actual level of blood safety achieved in routine practice. This study aimed to estimate the residual risk of HIV, HBV, and HCV transmission among blood donors in the Borgou region between 2023 and 2025. A total of 11,113 repeat donors were analyzed, representing a well-defined population for incidence-based estimation.

### 4.1 Sociodemographic profile of donors

The donor population was predominantly male, with a sex ratio of 14.14, reflecting a persistent gender imbalance commonly reported in sub-Saharan Africa. Similar findings have been described in Mali and the Democratic Republic of Congo, where male dominance among donors is also pronounced (16, 17). This imbalance may be explained by physiological and medical deferrals among women, particularly related to pregnancy, breastfeeding, and anemia, but also by sociocultural factors that often position blood donation as a male-dominated activity.

The predominance of young donors, particularly those aged 18–30 years and students, reflects recruitment strategies centered on educational institutions. This age group is generally considered suitable for donation due to better perceived health status and accessibility. However, this demographic concentration may also introduce epidemiological vulnerability, as young adults may engage in higher-risk behaviors while having incomplete vaccination coverage, particularly against hepatitis B. The predominance of mobile collection teams highlights the importance of outreach strategies in maintaining the blood supply. However, this approach may also limit the recruitment of regular donors, as it often favors opportunistic rather than sustained donation behavior.

### 4.2 Donation frequency and donor retention

The relatively low frequency of donations observed in this study indicates a predominance of occasional donors, with half donating only twice per year. This pattern suggests limited donor retention and heavy dependence on mobile campaigns. The irregular availability of mobile teams, combined with donor mobility (especially students during academic breaks), likely contributes to inconsistent donation patterns. These findings emphasize the need to strengthen donor retention strategies and promote fixed-site donation systems to ensure a more stable and predictable blood supply.

### 4.3 Incidence of HIV, HBV, and HCV

A total of 145 incident infections were identified, with HBV accounting for the majority of cases. This predominance is consistent with the high endemicity of HBV in West Africa and its well-documented elevated prevalence among blood donors (18). The higher burden of HBV compared to HIV and HCV reflects both its greater transmissibility and its higher background prevalence in the general population.

The incidence rates observed in this study are substantially lower than those reported in Burkina Faso and the Democratic Republic of Congo, where much higher rates have been documented (19–20, 27–29). These differences may reflect improvements in donor selection, better implementation of screening strategies, and increased awareness among donors in Benin. However, despite this relative improvement, the observed incidence remains significantly higher than that reported in high-income countries such as France, where incidence rates are extremely low due to advanced screening technologies and low population prevalence (21).

### 4.4 Sociodemographic determinants of infection

No significant association was observed between HIV incidence and sociodemographic variables, suggesting a more generalized exposure pattern. However, the interpretation of HIV related findings is limited by the very small number of cases. In contrast, HBV and HCV infections showed a significant association with younger age groups, particularly 18–24 and 25–30 years. This may reflect behavioral and epidemiological factors, including higher levels of sexual activity, incomplete HBV vaccination coverage, and increased exposure risk in densely social environments such as universities.

The higher incidence observed among donors recruited through mobile teams may be explained by the predominance of young student donors in this group, who are typically occasional donors and may not be fully integrated into structured donor follow-up systems. These findings highlight the importance of targeted prevention strategies, including HBV vaccination campaigns in schools and universities, as well as improved health education focused on risk reduction.

### 4.5 Residual transfusion risk

The residual risk of transfusion-transmitted infections in Borgou was estimated at 1 in 277,000 donations for HIV, 1 in 1,862 for HBV, and 1 in 7,092 for HCV. The highest residual risk was observed for HBV, which is consistent with its higher incidence and the biological complexity of infection, including long window periods and the possibility of occult hepatitis B infection.

These findings are consistent with studies conducted in the Democratic Republic of Congo, which also identified HBV as the principal contributor to residual transfusion risk (20, 22). However, the residual risks observed in this study are lower than those reported in several West African countries such as Burkina Faso, Côte d’Ivoire, and Senegal, where significantly higher estimates have been documented (19, 23, 24).

This relative reduction in residual risk in Benin may be attributed to several factors, including strict donor selection criteria, exclusive reliance on voluntary non-remunerated donors, and the use of high-quality serological screening assays. These elements contribute to improving transfusion safety, although they remain insufficient to eliminate risk.

In contrast, residual risks in developed countries are several orders of magnitude lower. This difference is largely explained by the low prevalence of transfusion-transmissible infections in the general population, strict donor deferral policies, and the widespread use of nucleic acid testing (NAT), which significantly reduces the serological window period and enhances early detection of infections (21, 25). As demonstrated by Rerambiah et al. (26), the implementation of NAT substantially improves blood safety by detecting infections during the pre-seroconversion phase.

### 4.6 Limitation of the study

This study has several methodological limitations. The use of the incidence/window period model is an indirect approach that relies on window period estimates derived from the literature. Although widely used in transfusion medicine, the absence of molecular screening (TAAN/NAT) may lead to an underestimation of the residual risk, particularly for infections in the very early stage.

In addition, restricting the analysis to regular donors may introduce a representativeness bias of the overall donor population. Furthermore, the limited consideration of biological and pre-analytical variability, as well as the very small number of HIV cases, restricts the scope and generalizability of the findings. Finally, the lack of data on risk behaviors (sexual practices, exposure to invasive procedures, and hepatitis B vaccination status) limits the analysis of factors associated with infection incidence.

## 5 Conclusion

This study provides a comprehensive evaluation of the residual risk of transfusion-transmitted HIV, HBV, and HCV infections in the Borgou region of Benin over a three-year period among 11,113 repeat blood donors. The findings reveal low but persistent residual risks, with HBV as the leading contributor, followed by HCV and HIV. A higher burden of HBV and HCV was observed among young donors aged 18–30 years and among individuals recruited through mobile collection campaigns, highlighting the influence of demographic structure and outreach-based recruitment strategies on donor risk profiles. Although these residual risks are lower than those reported in several sub-Saharan African settings, they remain markedly higher than those observed in high-income countries, underscoring persistent structural disparities in transfusion safety linked to infection prevalence and diagnostic capacity.

From a public health perspective, these results emphasize the need to strengthen blood safety strategies through improved donor selection and retention, with a focus on maintaining a stable pool of regular low-risk voluntary donors. Targeted prevention strategies, including health education and systematic hepatitis B vaccination among adolescents and young adults, are essential to reduce ongoing transmission within the donor population. Ultimately, the integration of nucleic acid testing (NAT) into existing screening algorithms, alongside enhanced donor management strategies, represents a critical step toward minimizing the residual risk and achieving a safer and more resilient blood transfusion system in Benin.

## Data Availability

The raw data supporting the conclusions of this article will be made available by the authors, without undue reservation.
